# Evolution of the Kondo lattice and non-Fermi liquid excitations in a heavy-fermion metal

**DOI:** 10.1038/s41467-018-05801-5

**Published:** 2018-08-20

**Authors:** S. Seiro, L. Jiao, S. Kirchner, S. Hartmann, S. Friedemann, C. Krellner, C. Geibel, Q. Si, F. Steglich, S. Wirth

**Affiliations:** 1 Max Planck Institute for Chemical Physics of Solids, 01187 Dresden, Germany; 2 Zhejiang Institute for Modern Physics, Zhejiang University, 310027 Hangzhou, PR China; 3 Helmholtz-Zentrum Dresden-Rossendorf, 01328 Dresden, Germany; 4 School of Physics, University of Bristol, Bristol, BS8 1TH UK; 5 Institute of Physics, Goethe-University Frankfurt, 60438 Frankfurt/Main, Germany; 6 Department of Physics and Astronomy, Rice University, Houston, TX 77005 USA; 7 Present Address: Institute for Solid State Physics, IFW-Dresden, Helmholtzstrasse 20, 01069 Dresden, Germany

## Abstract

Strong electron correlations can give rise to extraordinary properties of metals with renormalized Landau quasiparticles. Near a quantum critical point, these quasiparticles can be destroyed and non-Fermi liquid behavior ensues. YbRh_2_Si_2_ is a prototypical correlated metal exhibiting the formation of quasiparticle and Kondo lattice coherence, as well as quasiparticle destruction at a field-induced quantum critical point. Here we show how, upon lowering the temperature, Kondo lattice coherence develops at zero field and finally gives way to non-Fermi liquid electronic excitations. By measuring the single-particle excitations through scanning tunneling spectroscopy, we find the Kondo lattice peak displays a non-trivial temperature dependence with a strong increase around 3.3 K. At 0.3 K and with applied magnetic field, the width of this peak is minimized in the quantum critical regime. Our results demonstrate that the lattice Kondo correlations have to be sufficiently developed before quantum criticality can set in.

## Abstract (web summary)

The competition between Kondo correlations and magnetic ordering in heavy fermion materials leads to emergent physics that is not fully understood. Seiro et al. show inter-site Kondo correlations only dominate over local ones at temperatures well below the single-ion Kondo temperature but this is a prerequisite for quantum critical behavior.

## Introduction

Heavy fermion materials, i.e. intermetallics that contain rare earths (REs) like Ce, Sm, and Yb or actinides like U and Np, are model systems to study strong electronic correlations^1,2^. The RE-derived localized 4*f* states can give rise to local magnetic moments which typically order (often antiferromagnetically) at sufficiently low temperature as a result of the inter-site Ruderman–Kittel–Kasuya–Yosida interaction. In addition, the on-site Kondo effect causes a hybridization between the 4*f* and the conduction electrons, which eventually screens the local moments by developing Kondo spin-singlet many-body states. Hence, these two interactions directly compete with each other and lead to different (long-range magnetically ordered vs. paramagnetic Fermi-liquid) ground states^3^. A zero-temperature transition between the two states can be controlled through doping, pressure or magnetic field *H*. A quantum critical point (QCP) and concomitantly non-Fermi liquid properties ensue if the phase transition is continuous at zero temperature^4–6^.

Heavy fermion metals have been established as a canonical setting for quantum criticality^2^. How the Kondo lattice coherence develops upon lowering the temperature, i.e. the hierarchy of energy scales, is, however, still a matter of debate. Intuitively, the coherence temperature *T*_coh_ is set by the single-ion Kondo temperature *T*_K_ of the lowest-lying crystal-field level^7^ and can be further reduced by disorder^8^, while within another model *T*_coh_ can exceed *T*_K_ considerably^9,10^. The latter model might be related to the influence of crystalline electric field (CEF) effects^2,11^. Considerable experimental efforts have recently been devoted to the study of the quantum critical regime at sufficiently low temperatures. A key observation is that quantum criticality induces a large entropy, suggesting that it is linked with the Kondo effect. This raises the important question^12^ as to how the onset of Kondo lattice coherence at elevated temperatures connects with the emergence of quantum criticality at low temperatures.

**Fig. 1:**
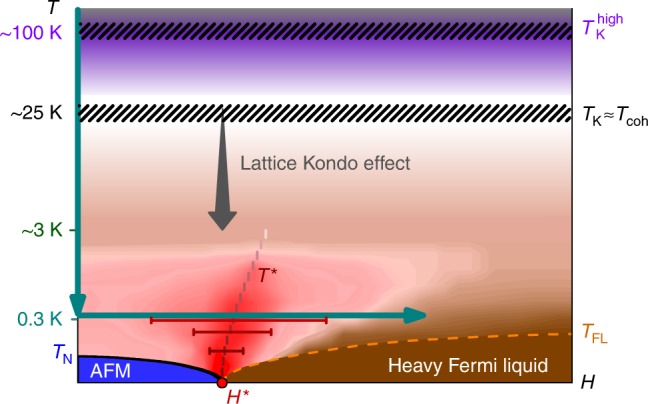
Phase diagram of YbRh_2_Si_2_. Schematic temperature–magnetic field phase diagram as inferred from magnetotransport^15,16^, magnetostriction^17^, and magnetization^20^ measurements at low *T*, and STM measurements down to ~5 K^7^. The QCP (red dot) is located at H^*^ ≈ 0.06 T for *H*⊥*c* and H^*^ ≈ 0.66 T for $H||c$. The single-ion Kondo temperatures $T_{\mathrm{K}}^{{\mathrm{high}}}$ and *T*_K_ involve all (purple shading) and the lowest-lying (white) crystal electric field levels, respectively. The lattice Kondo effect starts to develop around *T*_coh_ ≈ *T*_K_. The Kondo-exchange interaction between the two types of spins, respectively, belonging to the local moments or the conduction electrons, gives rise to Kondo correlations in the spin-singlet channel, which are always dynamical at finite temperatures. The lattice Kondo effect (gray arrow) grows as temperature is decreased. At large magnetic fields, lowering the temperature eventually turns the short-lived lattice Kondo correlations into long-lived ones (brown region) indicating a heavy Fermi liquid with renormalized (large) Fermi surface well below *T*_FL_. For small magnetic fields the correlations stay dynamical. Here, an antiferromagnetic (AFM) order (blue region) develops below the Néel temperature *T*_N_, again with long-lived lattice Kondo correlations. The reddish regime embedding the T^*^(*H*) crossover line indicates incoherent quantum critical fluctuations as the system evolves towards the respective ground state on either side^15–17^. This scale, anchored by the QCP, marks the finite-temperature signature of the Mott-type phase transition at *T* = 0, additionally visualized by the red bars corresponding to the width of the crossover in Hall effect^16^. The green arrows indicate the parameters used in STS measurements

The prototypical heavy fermion metal YbRh_2_Si_2_ shows an antiferromagnetic (AFM) ground state with a very low Néel temperature, *T*_N_ = 70 mK, and a QCP upon applying a relatively small field *μ*_0_*H*_N_ = 0.66 T parallel to the tetragonal *c*-axis. Non-Fermi liquid behavior has been observed in the quantum critical regime (i.e. at finite field), extending up to temperatures of about 0.5 K^13^, depending on the physical quantity that is measured as well as the degree of disorder^14^, see *T*–*H* phase diagram in Fig. 1. Isothermal magnetotransport^15,16^ and thermodynamic^17^ measurements at low temperatures have provided evidence for the existence of an additional low-energy scale T^*^(*H*), which has been interpreted as the finite-temperature manifestation of the critical destruction of the lattice Kondo effect^18^ and the concomitant zero-temperature jump of the Fermi surface from large to small across the QCP. Measurements of the thermal and magnetic Grüneisen ratio strongly support this picture^19,20^. An ever pressing issue, however, is the huge specific heat coefficient even in zero magnetic field^14,21^, which implies an abundance of fluctuations. Below *T*_N_, these are of Fermi-liquid type. Above *T*_N_, an obvious cause of these fluctuations are dynamical Kondo correlations, and above ~0.5 K YbRh_2_Si_2_ at zero field belongs to the quantum-critical fluctuation regime^13^. Yet, alternative scenarios have been proposed as well^22–24^.

Scanning tunneling spectroscopy (STS) measures locally the density of states (DOS)^25^ through single-particle excitations^7,26,27^. Spectra obtained at temperatures *T* ≥ 4.6 K and *H* = 0 revealed the successive depopulation of the excited CEF states as the temperature is lowered, with essentially only the lowest crystal-field Kramers doublet occupied at lowest temperatures^7^. The coupling between the localized 4*f* electrons in this Kramers doublet and the conduction electrons gives rise to periodic Kondo-singlet correlations which start to develop below *T*_coh_. This coherence temperature is linked to the effective single-ion Kondo temperature *T*_K_ ≈ 25 K extracted from bulk measurement^28^. While these properties conform to the traditional understanding of the high-temperature behavior of the Kondo lattice^29,30^, the questions remain open on how the Kondo coherence evolves further upon lowering the temperature^13,31,32^ and in applied field (green arrows in Fig. 1) and, importantly, how it connects with quantum criticality.

We therefore measure STS down to 0.3 K and in applied magnetic fields up to 12 T, complemented by magnetotransport and thermopower measurements on identical YbRh_2_Si_2_ samples. We find that lattice Kondo correlations dominate only at temperatures about an order of magnitude below the single-ion Kondo temperature. Substantial lattice Kondo correlations are a prerequisite for quantum criticality to set in.

## Results

### Temperature evolution of tunneling spectra down to 0.3 K

**Fig. 2:**
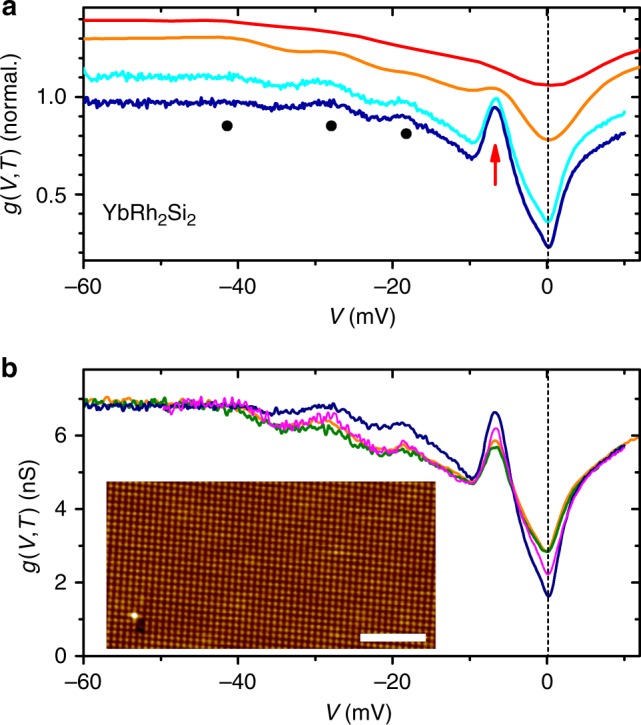
Tunneling spectroscopy on YbRh_2_Si_2_. **a** Tunneling conductance *g*(*V*, *T*) normalized at *V* = −80 mV and obtained at 0.3, 1.7, 5.5, and 30 K (from bottom to top), curves at 1.7, 5.5, and 30 K are offset for clarity. The −6 meV-peak evolves strongly at low *T* (red arrow). Black dots mark features resulting from CEF splitting of the Yb 4*f* multiplet. **b** *g*(*V*, *T*)-curves at selected low *T* (blue: 0.3 K, magenta: 1 K, orange: 1.9 K, olive: 3.3 K) obtained on the Si-terminated surface shown in the inset. Inset: Topography indicating excellent surface quality (scale bar: 4 nm, *V* = 100 mV, *I* = 0.6 nA)

Tunneling conductance curves d*I*/d*V* = *g*(*V*,*T*) obtained over a wide range of temperatures are presented in Fig. 2a. Both, the peaks due to CEF splitting of the Yb^3+^ multiplet (marked by black dots in Fig. 2a) and the conductance dip at zero bias (*V* = 0), result from single-ion Kondo physics^7^. Specifically the latter signifies the hybridization between 4*f* and conduction electrons. The most striking feature, however, is the evolution of the peak at about −6 mV (red arrow in Fig. 2a). This peak initially develops below 30 K, but clearly dominates the spectra only for $T \lesssim 3.3\,{\mathrm{K}}$.

We now focus on this low-temperature regime *T* ≤ 3.3 K (Fig. 2b). These data were obtained on the surface shown in the inset where topography over an area of 20 × 10 nm^2^ is presented. Such a topography not only attests the excellent sample quality but is also indicative of Si termination (see Supplementary Note 1 and Supplementary Figs. 1, 2). This termination is pivotal to our discussion as it implies predominant tunneling into the conduction electron states. A hint toward the origin of the −6 mV-peak comes from renormalized band structure calculations^33^: a partially developed hybridization gap is seen in the quasiparticle DOS at slightly smaller energy. Here, as a result of the renormalization the 4*f* band is shifted close to the Fermi level. Since tunneling spectroscopy on Si-terminated surfaces primarily probes conduction electrons and the total number of electrons must remain constant, the hybridization gap in the 4*f*-band is seen as a peak in our tunneling spectroscopy. On the other hand, a multi-level, finite-U non-crossing approximation (NCA) described our temperature-dependent tunneling spectra away from the energy range of this peak reasonably well^7^ but presented no indication for the existence of a peak at −6 mV. Since NCA does not include intersite Kondo correlations it is very reasonable to assume that this peak results from a strong development of lattice coherence, i.e. the lattice Kondo effect, and will be referred to as the Kondo lattice peak. The bulk nature of the −6 mV peak is supported by comparison to bulk transport measurements, as discussed below.

**Fig. 3:**
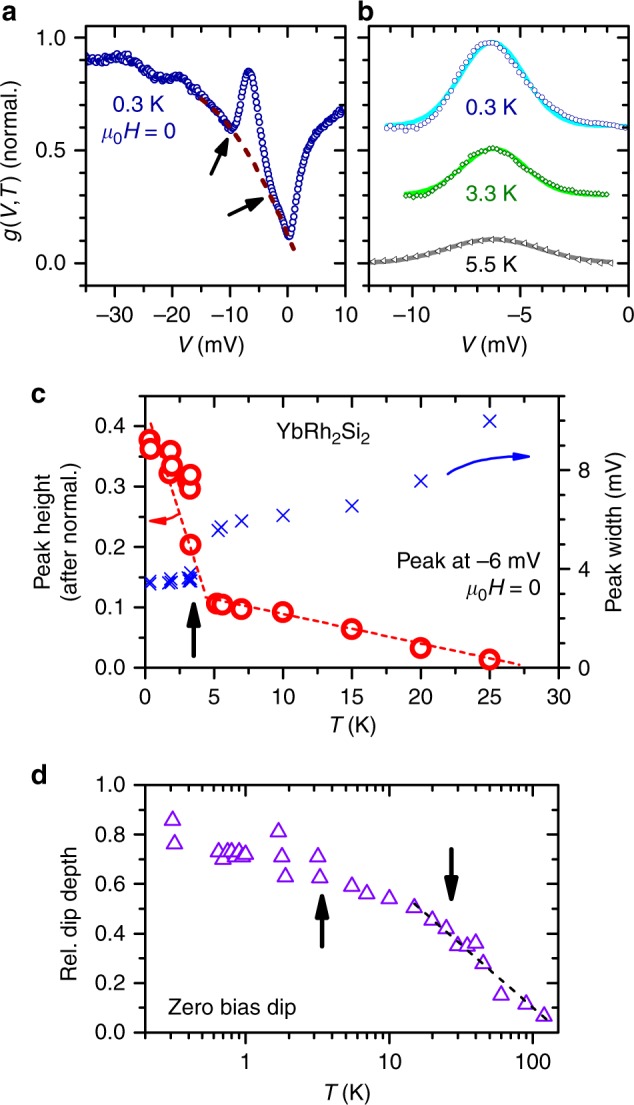
Analysis of the Kondo lattice peak. **a** Tunneling conductance *g*(*V*, *T* = 0.3 K) normalized to its value at *V* = −80 mV, and parabola used for background subtraction (dashed line). Arrows indicate onset of deviations between data and parabola. **b** Examples of *g*(*V*, *T*, *H* = 0)-data after background subtraction (hollow markers, data sets at *T* < 5.5 K are offset). Data can be well described by Gaussians (lines). **c** Height (circles) and width (FWHM, crosses) of the peak at −6 mV after normalizing all *g*(*V*, *T*)-curves at −80 mV. At *T*_P_, indicated by the upward arrow, peak height and width change significantly. Results from different samples cause several markers to overlap. Dashed lines are guides to the eye. **d** Relative depth of the single-ion Kondo dip at zero-bias. Low-*T* data were obtained on several surfaces of two different samples, data at *T* ≥ 5 K from ref.^7^. The upward arrow indicates *T*_P_ (as in **c**), the downward arrow *T*_K_. Dashed line is a logarithmic fit to the data as proposed in ref.^34^

An analysis of the Kondo lattice peak is impeded by the strongly temperature-dependent zero-bias dip close by (see also Fig. 3d, Supplementary Notes 2, 3 and Supplementary Figs. 3, 4). Data $g( V,T \,\gtrsim \, 30\,{\mathrm{K}} )$ for −15 mV ≤ *V* ≤−3 mV can be well approximated by a parabola and hence, we assume a parabola to describe the background below the Kondo lattice peak at low temperature, see the example of *T* = 0.3 K in Fig. 3a. There are finite energy ranges on both sides of the peak feature allowing to fit a parabola, cf. arrows in Fig. 3a. After background subtraction, each peak can be well described by a Gaussian (lines in Fig. 3b) from which its height and width (full width at half maximum, FWHM) is extracted. Note that the peak position in energy is independent of temperature (Fig. 3b). Clearly both, the peak height and FWHM, exhibit a significant change across *T*_P_ ≈ 3.3 K, Fig. 3c. In contrast, the dip in zero-bias conductance, the hallmark of the single-ion Kondo effect, smoothly continues to deepen (Fig. 3d, for data on linear *T*-scale see Supplementary Fig. 4). Here, the depth of the zero-bias dip is defined as $1 - [g(V = 0,T)/g(V = - 80\,{\mathrm{mV}},T)]$^7^. This depth decreases logarithmically for 10 K < *T* ≤ 120 K, i.e. around *T*_K_, as predicted by dynamical mean field theory^34^.

### Comparison to magnetotransport and thermopower measurements

**Fig. 4:**
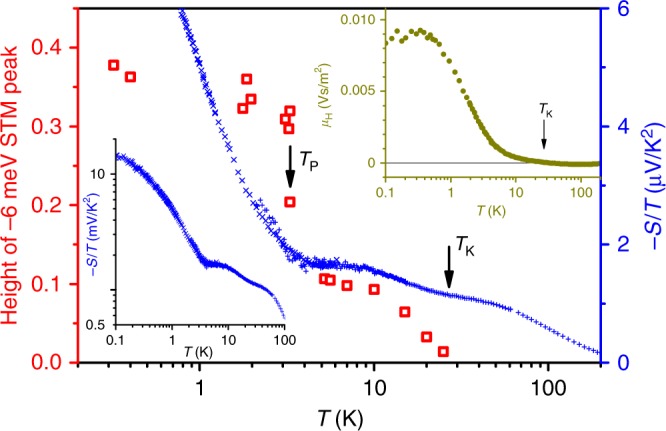
Development of lattice Kondo correlations. The height of the Kondo lattice peak (red squares) is compared to thermopower *S* divided by *T* (blue crosses) in dependence on *T*. Low-temperature data (*T* ≤ 6 K) were taken from ref.^35^. Left inset: same *S*/*T*-data on a logarithmic scale to show broader range. Right inset: Hall mobility *μ*_H_ vs*. T*. All three properties exhibit a strong upturn below *T*_P_ ≈ 3.3 K and saturation at lowest *T*

While this temperature evolution of the single-particle spectrum is surprising, it connects well with the features that appear in bulk transport measurements^14–17,35,36^. Importantly, Fig. 4 shows that the thermopower divided by temperature, −*S*/*T*, has a qualitatively similar temperature dependence as the height of the STS Kondo lattice peak. Both display a plateau below about 7 K, and a subsequent strong increase upon lowering the temperature below *T*_P_ ≈ 3.3 K. Here, *T*_P_ is defined as the temperature at which the −6 meV-peak strongly develops. In the zero-temperature limit, a Fermi liquid is characterized by a constant value *S*/*T*. For a Kondo lattice system, this is expected to be seen at very low temperatures, i.e., once the renormalized band structure is almost fully developed^37^. In fact, for YbRh_2_Si_2_ heavy Fermi-liquid behavior was observed beyond the QCP: At fields *μ*_0_*H* = 1 T, the coefficient −*S*/*T* reaches ≈ 7 μV/K^2^ for temperatures up to ~0.5 K^35^, indicative of a very large effective charge carrier mass. The plateau in *S*/*T* seen in Fig. 4 occurs at a value almost an order of magnitude smaller and extends to a correspondingly higher temperature (see also Supplementary Note 5). This indicates some medium heavy Fermi liquid, i.e. prevailing Kondo-lattice correlations. Moreover, the nearly logarithmic increase in *S*/*T* below *T*_P_ resembles that of the Sommerfeld coefficient *γ* of the electronic specific heat^14^ and is a clear signature of non-Fermi liquid behavior^35^. Therefore, the comparison of our STS results with those of *S*/*T* naturally leads us to propose that the incipient saturation of the Kondo lattice peak height below about 7 K (Fig. 4) signifies some prevailing Kondo–lattice correlations and, importantly, the growth of this peak below *T*_P_, as well as the concomitant drop of peak width (Fig. 3c) capture the quantum critical behavior. This leads to the insight that quantum criticality arises not before there is sufficient buildup of lattice Kondo correlations (see Supplementary Note 4 and Supplementary Fig. 5), or conversion of the local 4*f* electron spins into extended quasiparticle-like, but still incoherent excitations.

To illustrate this point further, the Hall mobility *μ*_H_ = *R*_H_/*ρ*_*xx*_ as a function of temperature is also plotted in Fig. 4, right inset (*R*_H_ itself is compared to *S*/*T* in Supplementary Fig. 6). In the regime where the anomalous Hall effect dominates, this quantity has been considered as capturing the buildup of the on-site Kondo resonance^38^. It is striking that the Hall mobility also shows a strong increase upon lowering the temperature. Yet, the Hall mobility does not show any plateau near 3 K, and neither does the resistivity nor the Sommerfeld coefficient as a function of temperature^14^. This implies that *T*_P_ ≈ 3.3 K is not an ordinary Fermi liquid scale. The connection between the growth of the Hall mobility with quantum criticality becomes evident when we analyze its inverse 1/*μ*_H_ = *ρ*_*xx*_/*R*_H_, which is equivalent to the cotangent of the Hall angle, cot *θ*_H_, as a function of temperature. In YbRh_2_Si_2_, a power-law behavior of 1/*μ*_H_, more specifically 1/*μ*_H_ ~ *T*^2^, is observed for 0.5 K $\lesssim T \lesssim$ 5 K (see Supplementary Fig. 7). Such a behavior, as well as the *T*-linear electrical resistivity seen in relevant parts of the phase diagram of YbRh_2_Si_2_^14^, has also been observed, e.g., in the cuprate high-*T*_c_ superconductors^39^.

### Evolution of tunneling spectra in magnetic fields

**Fig. 5:**
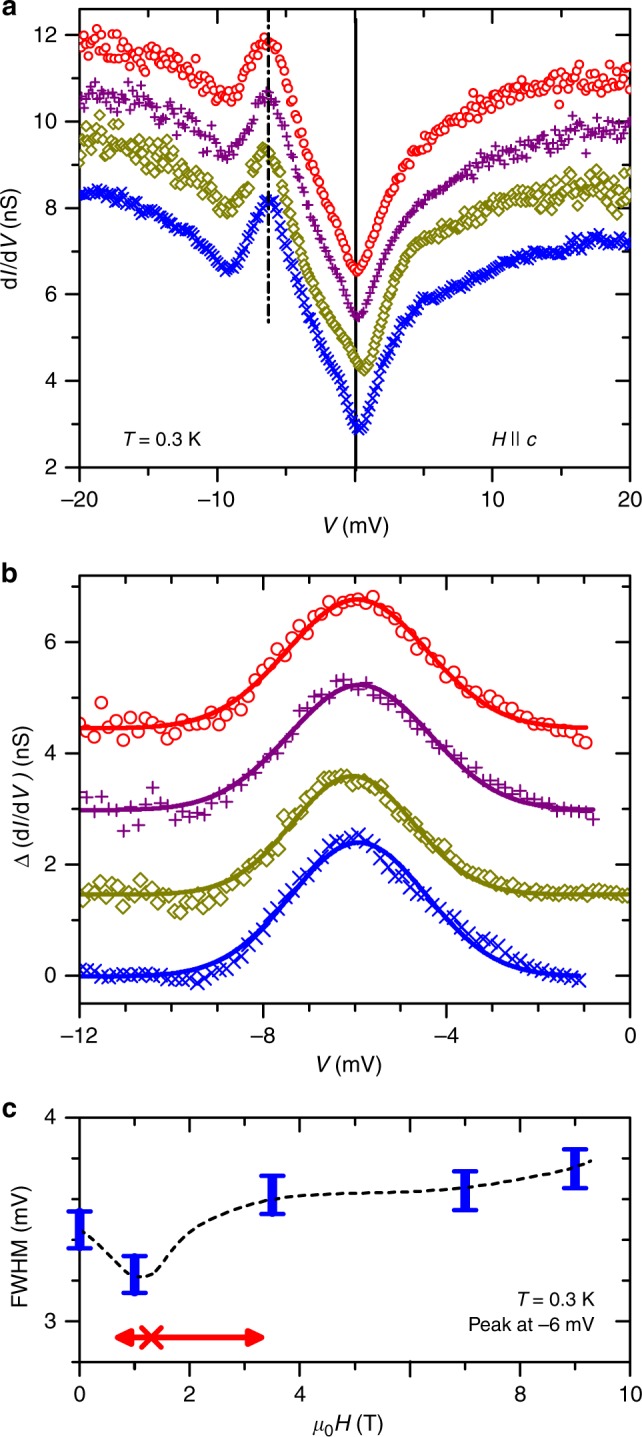
Spectroscopy in applied field. **a** Tunneling conductance *g*(*H*, *T* = 0.3 K) measured at different magnetic fields (0, 1, 7, 11 T from bottom to top) applied parallel to the magnetically hard *c*-axis. Curves are offset for clarity. **b** Tunneling conductance data of **a** after parabolic background subtraction (markers) as described in Fig. 3a. Lines are the corresponding Gaussian fits; fields and color scheme as in **a**. **c** FWHM of the Kondo lattice peak for different magnetic fields at *T* = 0.3 K. At this temperature and field orientation, the energy scale T^*^ (cf. Fig. 1) is located at a field of about 1.3 T (red cross^15,17^), approximately where a minimum is observed in the peak width. Several samples/cleaves were used to establish the existence of this minimum. The red arrow indicates the FWHM of the Hall crossover at *T* = 0.3 K ^16^. Height of blue error bars correspond to the errors of the Gaussian fits (Figs. 3a and b) and differences between samples, the line is a guide to the eye

To search for more direct STS evidence for quantum criticality in the *H*–*T* phase diagram of YbRh_2_Si_2_, the system was tuned by a magnetic field at *T* = 0.3 K ≈ 0.1*T*_P_, i.e. where coherent lattice effects are clearly dominating. Some *g*(*V*, *H*, *T* = 0.3 K)-curves are presented in Fig. 5a. No major change in the overall shape of the spectra with magnetic field is observed. The Kondo lattice peak can again be described by a Gaussian after parabolic background subtraction (Fig. 5b). Within the energy resolution of our STM the peak’s position in energy is independent on *H*. The resulting FWHM of the peak in dependence on *H* is presented in Fig. 5c. We note that the FWHM at low *T* and fixed *H* varies very little between different spectra, and even different samples, i.e. <4% (see also Fig. 3c where several data points of the FWHM fall on top of each other). This is taken as the error of FWHM, and determines the size of the error bars in Fig. 5c. Moreover, a comparison between the data and the Gaussian fit in Fig. 5b reveals an only slightly enhanced noise of *g*(*V*, *H*, *T* = 0.3 K) at elevated fields compared to zero field. Consequently, the trend displayed in Fig. 5c appears genuine.

At a field of *μ*_0_*H* = 1 T, the Kondo lattice peak FWHM exhibits a minimum, with a reduction of about 15% of its high-field value. This field is approximately of the value *μ*_0_H^*^ ≈ 1.3 T at which the Hall crossover takes place at *T* = 0.3 K for *H*||*c* (red cross in Fig. 5c, for the field direction see Supplementary Note 6). The range in magnetic field over which the Hall crossover is observed^16^ is indicated by a red arrow in Fig. 5c. This implies that changes in *g*(*V*, *H*, *T* = 0.3 K) are to be expected within a similar field range, as indeed suggested by the drop in peak width vs*. H* at *T* = 0.3 K (see also Supplementary Note 7 and Supplementary Fig. 8). Note that at this low temperature Kondo lattice effects are dominating. In this regime, the observed drop of peak width at *μ*_0_*H* = 1 T indicates a reduced quasiparticle weight and follows the expected behavior for a critical slowing down concluded from isothermal magnetotransport (Hall coefficient, *R*_H_, and magnetoresistance, *ρ*_*xx*_) measurements^15,16^, revealing thermally broadened jumps at H^*^(*T*). One may therefore expect that the drop in peak width may further increase and sharpen upon cooling, (cf. Fig. 1). In this view, all our findings reflect the finite temperature remnant of a field-induced QCP at *T* = 0. Data from specific-heat measurements on YbRh_2_Si_2_ in magnetic field^40^ confirm this assignment (cf. Supplementary Fig. 8). They yield a relative change of the Sommerfeld coefficient between critical (H^*^) and elevated fields of order 30% at *T* = 0.3 K, if scaled for the relevant field orientation. We believe that the larger change in Sommerfeld coefficient compared to the drop in FWHM of the STS Kondo lattice peak (Fig. 5c, about 15% compared to the value at 9 T at which YbRh_2_Si_2_ is almost in the Fermi liquid regime^41^) is related to the fact that heat capacity integrates over the whole Brillouin zone while STS is a more directional measurement. For a surface along the *a*–*b* plane (Fig. 2), tunneling along the *c*-direction is most relevant, yet hybridization of the Yb CEF ground state orbitals is anisotropic^33^, mostly with the Rh $4d_{x^2 - y^2}$.

Remarkably, the FWHM at zero field falls in line with its trend at high fields *μ*_0_*H* ≥ 3.5 T, i.e. there is no significant difference at *T* = 0.3 K at both sides of the QCP. While the presented STS data on its own do not allow to distinguish between quantum critical scenarios, they are in good agreement with isothermal magnetotransport data. Even at a temperature as low as ~0.5 K, the Hall crossover is expected to reach all the way to *H* = 0^13^. In analogy, the peak width in STS at *H* = 0 should be close to the one extrapolated from higher fields, where a large Fermi surface constitutes the heavy Fermi liquid. Crossing the T^*^-line at temperatures as high as about half a K, there is still a dominating contribution of the large Fermi surface to the quantum-critical fluctuations even at zero field^42^. Upon cooling, this contribution of the large Fermi surface at *H* = 0 is expected to decrease^13^. To establish this trend further, lower temperatures for our STS measurements are clearly called for. We note that Lifshitz transitions and Zeeman splitting can be ruled out as origins for the drop of the peak’s FWHM (see Supplementary Note 5).

## Discussion

Our STS studies here have revealed two important insights. One is that the development of the dynamical lattice Kondo correlations in a stoichiometric material such as YbRh_2_Si_2_, while setting in at *T*_coh_ ≈ *T*_K_, extends to considerably lower temperatures and dominate the material’s properties only at much lower temperatures (see Supplementary Note 4). In the case of YbRh_2_Si_2_, the STS Kondo lattice peak height and thermopower coefficient do not indicate dominant lattice Kondo correlations before the temperature has reached *T*_P_ ~ 0.1·*T*_coh_. Moreover, the conductance minimum at zero bias, which has been shown to capture primarily the on-site Kondo (i.e. hybridization) effect at temperatures $T \gtrsim 5\,{\mathrm{K}}$^7^, also continues to deepen down to the lowest measured temperature as shown in Fig. 3d. Conversely, the strengthening of the lattice Kondo coherence only at much below *T*_K_ implies that the on-site Kondo effect dominates many thermodynamic and transport properties at around and below *T*_coh_ in YbRh_2_Si_2_, and gives way to the lattice Kondo correlations only slowly upon reducing the temperature. Such a persistence of this distinct signature of the single-ion Kondo effect down to temperatures substantially below *T*_coh_ is consistent with observations based on different transport^37,38^ and thermodynamic^8,43^ properties of several other heavy-fermion metals. On the one hand, this provides a natural explanation to the applicability of single-ion-based descriptions to temperatures well below *T*_K_ even though they neglect lattice Kondo coherence effects^7,37,38^. On the other hand, this finding supports nicely the theoretical concept of two temperature scales, i.e. a single-ion and a lattice Kondo scale^29,30^, including the predicted order of magnitude difference^30^.

The second lesson concerns the link between the development of the dynamical lattice Kondo correlations and quantum criticality. As a function of temperature, our measurements of the height and width of the Kondo lattice peak strongly suggest that, in order for the quantum criticality to set in, the lattice Kondo correlations first have to develop sufficiently upon lowering the temperature through, and well below, *T*_K_ ≈ *T*_coh_ ≅ 30 K. More specifically, as the temperature is lowered through *T*_coh_, both the Kondo lattice peak height and the thermopower coefficient first reach a plateau below about 7 K signifying well-developed lattice Kondo correlations. It is against this backdrop that the Kondo lattice peak height and *S*/*T* markedly increase below *T*_P_ ≈ 3.3 K. This manifests quantum criticality at the level of the single-particle spectrum, which goes considerably beyond the quantum critical behavior seen in the divergent Sommerfeld coefficient of the electronic specific heat and the linear-in-*T* electrical resistivity^14^. This signature of the quantum criticality at the single-particle level is complemented by the isothermal behavior of the Kondo lattice peak with respect to the control parameter, the magnetic field, at the lowest measured temperature, *T* ≈ 0.3 K. The FWMH of this peak displays a minimum at a similar field value at which isothermal transport and thermodynamic measurements show a Fermi surface crossover^15–17^ indicating its relation to quantum criticality.

To put these findings into perspective, our comparative studies indicate an appealingly natural scenario: the development of the lattice Kondo correlations is the prerequisite for quantum criticality. Only if the Kondo lattice is sufficiently established quantum critical fluctuations can evolve. As such, the insights gained in our study will likely be relevant to the non-Fermi liquid phenomena in a broad range of other strongly correlated metals, such as the high-*T*_c_ cuprates and the organic charge-transfer salts, which are typically in proximity to Mott insulating states and in which quantum criticality is often observed^44–46^.

## Methods

### Sample characterization

High-quality single crystals of YbRh_2_Si_2_ were grown by an indium-flux method; they grow as thin platelets with a height of 0.2–0.4 mm along the crystallographic *c*-direction (see also Supplementary Note 6). Crystalline quality and orientation of the single crystals were confirmed by x-ray and Laue investigations, respectively. The residual resistivity *ρ*_0_ of the six samples investigated here ranged between 0.5 and 0.9 μΩ cm with no apparent differences in their spectroscopic results. The samples were cleaved in situ perpendicular to the crystallographic *c* direction at temperatures ~20 K. Subsequent to cleaving, the samples were constantly kept under ultra-high vacuum (UHV) conditions and did not exhibit any sign of surface degradation for at least several months, as indicated by STM re-investigation.

### Scanning tunneling microscopy and spectroscopy

STM and STS was conducted (using a cryogenic STM made by Omicron Nanotechnology) at temperatures between 0.3 and 6 K, in magnetic fields *μ*_0_*H* ≤ 12 T (applied parallel to the crystallographic *c* direction) and under UHV conditions (*p* < 2·10^−9^ Pa). Spectroscopic measurements were conducted using lock-in technique with *V*_rms_ = 0.2 mV. For the tunneling spectra shown, *g*(*V*, *T*)-data were averaged over areas of 1 × 1 nm^2^ on grids of 24 × 24. In zero magnetic field, the averaging area was repeatedly varied between zero (i.e. spectroscopy repeated at a given point) and 5 × 5 nm^2^ to ensure local homogeneity of the *g*(*V*,*T*)-data. For the temperature range 4.6 K ≤ *T* ≤ 120 K a second UHV STM (LT-STM) was utilized (*p* ≤ 3·10^−9^ Pa).

### Thermopower measurements

The thermopower *S* was measured by applying a temperature gradient to a rod-shaped sample of dimensions 4 × 0.5 × 0.1 mm^3^ out of the same batch as the samples used in STM/S measurements. For low temperatures 0.03 K $\lesssim T \lesssim$ 6 K, a home-built, dilution refrigerator-based setup was used, while measurements between 2 and 360 K were conducted in a PPMS (Quantum Design Inc.). The overlap of the two temperature ranges between 2 and 6 K serves as consistency check. Thermopower data in the high-temperature range compare nicely to those obtained earlier^47^. Hall effect measurements (see ref.^48^ for details) were conducted on the same sample as the thermopower measurements.

### Data availability

The data that support the findings of this study are available from the corresponding author upon request.

## Electronic supplementary material

Supplementary Information
