# Supplementary material for: Evolution of the Kondo lattice and non-Fermi liquid excitations in a heavy-fermion metal
Source: Nat Commun. 2018 Aug 20;9:3324. doi: 10.1038/s41467-018-05801-5 (PMC6102236; doi:10.1038/s41467-018-05801-5)
Supplement: Supplementary file 1 — Supplementary Information [file 41467_2018_5801_MOESM1_ESM.pdf]

Supplementary Information: Evolution of the Kondo lattice and non-Fermi liquid excitations in a heavy-fermion metal

S. Seiro, L. Jiao, S. Kirchner, S. Hartmann, S. Friedemann, C. Krellner, C. Geibel, Q. Si, F. Steglich, and S. Wirth

Supplementary Note 1:
Surface termination

We have cleaved a total of more than twenty single crystals $YbRh_2Si_2$. Out of those, 6 samples were cleaved *in situ* in our cryogenic STM, the remaining ones in our LT-STM (see Methods section). Both microscopes are equipped with identical cleaving stages and the resulting surfaces compare nicely.

Supplementary Fig. 1a shows topographies over a large surface area which make the following important aspects quite plain: i) Our cleaves of $YbRh_2Si_2$ so far resulted mostly in large, atomically flat terraces along the crystallographic *a*-*b* plane. The overall square alignment of the surface atoms as expected from the tetragonal structure, Supplementary Fig. 1b, is in excellent agreement with the lattice constant a = 4.007 Å, *cf.* Supplementary Fig. 1c. ii) The number of defects or impurities in the investigated samples is extremely small (10 per ∼3250 surface atoms for the example shown) which is in accord with the samples' small residual resistivities, *cf.* Methods section and Ref. 1. iii) There are mostly two types of defects, single protrusions of about 20 pm in additional height, and dumb-bell shaped dents of approximately 10–15 pm depth, Supplementary Fig. 1d. This is indicative of a Si-terminated surface[2,3]. Because of their excellent stability we concentrate on Si-terminated surfaces in our investigations.

Additional evidence in favour of Si-terminated surfaces stems from the CEF peak position: the energies of these peaks compare nicely to results from inelastic neutron scattering[4]. Hence, STS on such surface terminations allows to probe bulk properties, and the CEF peaks can conveniently be used to identify Si-terminated surfaces, in addition to topography discussed above.

In contrast, Yb-terminated surfaces exhibit single dents (40 pm and more in depth) in topography. In addition, Yb terminated surfaces typically exhibit two peaks in STS at shifted energy positions of −12 mV and −2 mV, possibly resulting from a surface state. Note, however, that all samples with possible Yb termination investigated so far showed less stable and less repeatable tunneling spectroscopy compared to Si terminated surfaces. To emphasize this point, Supplementary Fig. 2 displays examples of two spectra, obtained at different positions on a Yb terminated surface. Not unexpectedly, there are no indications of peaks at the energy positions of the CEF excitations, very likely because they are shifted in energy at the surface.

Here it is important to note that Yb within a Yb-terminated surface of $YbRh_2Si_2$ may exhibit a shift in valency compared to Yb within the bulk[5]. This effect can be seen even more drastically in $YbCo_2Si_2$ in which Yb is purely trivalent in the bulk, yet exhibits admixture of divalent states at the surface[6].

Supplementary Note 2:
Thermal smearing

Our measurements to very low temperature allow to clearly rule out that thermal smearing alone may account for the decay of the observed features in $g(V,T)$ with increasing temperature. As an example, the dashed line in Supplementary Fig. 3 represents the calculated data obtained by thermal smearing of the $g(V,T\!=\!0.3\,\mathrm{K})$-curve (blue) to 5.5 K, *i.e.* by evaluating $\int g(eV,T\!=\!0.3\,\mathrm{K})(df/dE)|_{(E-eV)}dE$. In this case, the Fermi function $f(T,E)$ is taken at T = 5.5 K. The so-calculated thermally broadened spectrum can directly be compared to the measured $g(V,T\!=\!5.5\,\mathrm{K})$ data (orange line in Supplementary Fig. 3). Clearly, much of the reduction of the −6 mV-peak and the depth of the zero bias dip is not thermal in origin.

Supplementary Note 3:
Alternative analysis of the Kondo lattice peak

The analysis employed to extract the height and FWHM of the Kondo lattice peak at −6 meV relies on a background subtraction and subsequent fitting of a Gaussian. In an effort to analyze our spectroscopy data fully independently of any background and/or fit, we show in Supplementary Fig. 4, red data points, the differences between the tunneling conductances at about −6 and 0 meV normalized to its sum, $\Delta = [g(V \simeq -6\ \mathrm{meV},T) - g(V \simeq 0,T)]/[g(V \simeq -6\ \mathrm{meV},T) + g(V \simeq 0,T)]$ (the exact energies were slightly adjusted, by less than ±0.2 mV, such that the maximum and minimum tunneling conductances, respectively, were captured). Because of the finite difference in energy of these two positions, the existence of the zero-bias dip keeps this difference at a finite value even when the peak at −6 meV has already vanished at T > 25 K. Nonetheless, this analysis clearly supports the change in slope of the temperature-dependent height of the Kondo lattice peak at $T \approx 5$ K $\gtrsim T_\mathrm{P}$, see dashed lines in Supplementary Fig. 4.

In this figure, we also show the relative depth of the zero-bias dip, *i.e.* after normalizing to $g(V\!=\!-80\,\mathrm{meV},T)$. The zero-bias dip does not, in line with our assignment to the on-site Kondo effect, provide an indication for a marked change at around 5 K. Hence, the clear increase in Δ below 5 K (*cf.* dashed line in Supplementary Fig. 4) is primarily

due to a changed Kondo lattice peak height rather then a considerably deepened zero-bias dip, supporting our analysis of the main text.

Supplementary Note 4: Dynamical lattice Kondo effect

The dynamical lattice Kondo effect refers to the dynamical correlations in the Kondo-singlet channel between the spins of the local moments and conduction electrons in a Kondo lattice system. Here we elaborate on this effect discussed in the main text and illustrated in Fig. 1 there.

We expound on the discussion through the results of an Extended Dynamical Mean Field Theory (EDMFT) study of a Kondo lattice[7]; further discussions can be found in Sec. VI of Ref. 8. The phase diagram is shown in Supplementary Fig. 5a, where I marks the RKKY interaction between the local moments, and T_K^0 the bare Kondo temperature. The tuning parameter is defined as $\delta \equiv I/T_K^0$.

For $\delta < \delta_c \approx 1.2$, the ground state is a heavy fermion state with static Kondo effect. In this regime, the temperature evolution of the system has the usual form. The Kondo-exchange interaction gives rise to dynamical correlations in the spin-singlet channel, which starts to develop at temperatures around T_K^0 and grows as the temperature is decreased. Lowering the temperature eventually turns the dynamical lattice Kondo effect into a static one, defined in terms of a nonzero static amplitude of the Kondo singlet. The strength of this Kondo singlet in the ground state is reduced as δ is tuned towards δ_c, and vanishes as δ_c is reached.

For $\delta > \delta_c$, an antiferromagnetic (AFM) order develops while the static Kondo effect is destroyed. Correspondingly, the Fermi surface is small. In this regime, the lattice Kondo effect, which is also initiated at temperatures around T_K^0, will stay dynamical down to $T = 0$. The fact that the dynamical lattice Kondo effect continues to operate in the AFM phase is illustrated by the calculation of the dynamical local spin susceptibility, $\chi_{\rm loc}$, as a function of the (Matsubara) frequency, ω_n, Supplementary Fig. 5b. As δ decreases from deep in the AFM ordered regime towards δ_c, the enhanced dynamical lattice Kondo effect is demonstrated by the gradual increase of the dynamical local spin susceptibility[7,9,10]. This effect is also manifested in the enhanced effective quasiparticle mass, which is a dynamical quantity describing the quasiparticle dispersion. This mass diverges as the QCP is approached from both the AFM and the paramagnetic (PM) sides.

Supplementary Note 5: Comparison to other measurements

To allow for better comparison, we also show results of thermopower measurements at an enlarged temperature scale, see Supplementary Fig. 6. Here, the change of slope of S/T just above 3 K is more clearly visible. At a similar temperature (within a factor of 2), the measured Hall coefficient $R_{\rm H}$ starts to deviate from a temperature independent value between 7 K $\lesssim T \lesssim$ 20 K (data taken from Ref. 11). Also, below about 1 K the anomalous contribution to $R_{\rm H}$ is negligible[12]. Hence, the additional contribution to $R_{\rm H}$ with maximum at $T \sim 0.7$ K is likely a result of the quantum critical fluctuations present in this temperature regime. We note that all these measurements, including our STM results reported in the main text, were obtained on samples of similar high quality, *i.e.* of residual resistivities $\rho_0 \leq 0.9\ \mu\Omega\,$cm.

Below 0.5 K, the Sommerfeld coefficient γ of the electronic specific heat, which shows a logarithmic increase upon cooling to below 10 K, turns over to some steeper power-law divergence[13]. The related phenomenon in the thermopower coefficient $S(T)/T$ is a peak around 0.1 K and a sign change in S/T at ∼30 mK[14]. Thus, the low-T $S(T)$ investigations of Ref. 14 reveal, in the zero-temperature limit, a change from $S/T > 0$ for $H < H_{\rm N}$ ($H_{\rm N}$ is the critical field) to $S/T < 0$ for higher fields. This sign change is in excellent agreement with experimental data[15] and theoretical results of renormalized band structure calculations[11], which illustrate that the Fermi surface at low fields contains two hole bands, while at elevated fields it is made up of one hole and one dominant electron band, *i.e.* there is a change from the so-called "small" to the "large" Fermi surface.

The inverse of the Hall angle, $1/\mu_H = \rho_{xx}/R_H = \cot\theta_H$, in dependence on temperature is presented in Supplementary Fig. 7. Here, the temperature is plotted as T^2 to emphasize the proportionality $1/\mu_H \sim T^2$ within the temperature range 0.5 K $\lesssim T \lesssim$ 5 K. In order to get an impression on the lower bound of this proportionality range, the inset provides a zoom into the temperature range below 1 K. We note that the magnetotransport measurements presented here were conducted on a sample out of the same batch from which also the sample for thermopower measurements and the majority of samples for the STM and STS measurements were obtained to allow for a comparison of the data as directly as possible.

Recently, thermopower, magnetoresistivity and Hall effect measurements up to fields of 15 T have been conducted[16,17] on single crystals of $YbRh_2Si_2$. These measurements, in concert with field-dependent renormalized band calculations, signaled Lifshitz transitions at around 3.4 T, 9.3 T and 11.0 T, with no indication for additional transitions at lower fields from either the measurements or the calculations. Moreover, both the Hall measurements and the renormalized band calculations show that the de-renormalization of the quasiparticles takes place continuously and requires magnetic fields of several tens of Tesla. Consequently, the f-electrons are driven out of the Fermi volume smoothly by an applied magnetic field of the order of the Kondo field H_K, way above the Fermi-surface crossover field H^* which signals the close proximity of the QCP.

Supplementary Note 6: Geometry considerations

As mentioned in the Methods section, application of the indium-flux method[1,18] resulted in samples grown as thin platelets. Therefore, it has not been possible so far to cleave a sample successfully along a plane other than the crystallographic *a*-*b* plane. Moreover, our STM setup presently requires scanning within a plane perpendicular to the magnetic field direction. Given these geometrical constrains all measurements within applied magnetic field were conducted with the field parallel to the crystallographic *c*-direction of our $YbRh_2Si_2$ samples. This direction constitutes the magnetically hard direction as, *e.g.*, seen in measurements of the magnetic susceptibility and the isothermal magnetization[19]. It should be noted that the ratio of the critical fields for the suppression of the antiferromagnetic order with magnetic fields applied within and perpendicular to the crystallographic *a*-*b* plane, respectively, is typically around 1/11, see Refs. 15, 20, and 21.

Supplementary Note 7: Width of the quasiparticle peak in STS

As pointed out in the main text, STM measures a local DOS in real space and hence, gives direct access to the number of states locally available to the system at a given energy interval, as well as their relaxation. Information on the Fermi momentum and thus, the Fermi volume is much harder to retrieve from STM spectra and requires further input (as *e.g.* in quasi-particle interference methods). The position of the -6 meV-peak is independent of the magnetic field strength which indicates that its changes in width reflect the field-dependence of the wave function renormalization and damping. We note that even deep in the Fermi liquid regime, momentum states beyond a quasi-particle description contribute to the local DOS whose relaxation rate does not follow a simple $(\boldsymbol{k}-\boldsymbol{k}_F)^2$-behavior at zero temperature.

When the tuning parameter, *i.e.* the magnetic field H, approaches H^*, we expect to see a critical slowing-down. Consequently, the isothermal width of the STS peak should develop a minimum, as is indeed experimentally observed (Fig. 5c of main text). More microscopically, when the quasiparticle residue, z, is only weakly dependent on the momentum, the width of the quasiparticle peak is linearly proportional to z. The zero-temperature limit of the $H^*(T)$-line is the Kondo-destruction quantum critical point, at which the quasiparticle residue $z = 0$. In other words, at $T = 0$, z is minimized at the critical field $H^*(T = 0)$. Correspondingly, at a non-zero temperature, the isothermal z will be minimized at the crossover field $H^*(T)$. Therefore the isothermal width of the STS peak will show a minimum at H^*.

It is instructive to compare the results we obtained for the width of the -6 meV-peak in applied magnetic field to those of the electronic contribution to the specific heat C_{el} at $T = 0.3$ K and in magnetic fields. Data of the latter for zero field[18] and in applied field[22] are plotted in Supplementary Fig. 8 as $C_{el}(H)/T$ *vs.* $\mu_0 H$ along with the data of Fig. 5c of the main text. Future measurements of both properties will have to show how closely the peak in $C_{el}(H)/T$ and the dip in FWHM(H) are separated from each other. Within the resolution of our current study they can be regarded as overlapping. This is in line with the Kondo-breakdown scenario[7,8] within which the smooth disappearance of the quasiparticle weight goes along with a divergence of the effective charge-carrier mass. Interestingly, as seen in Supplementary Fig. 8, the zero-field value of $C_{el}(H)/T$ exceeds the one at high fields. This reflects the surprising observation made previously[13] that the mass renormalization in the Fermi liquid which forms within the AF order at $H = 0$ is substantially stronger than in the heavy Fermi liquid within the paramagnetic phase at $H > H_N$ (where H_N is defined as the field at which $T_N \to 0$). Therefore, the specific heat, being a thermodynamic property and probing finite temperatures only, is enhanced dramatically within the Kondo-breakdown scenario by the dynamical Kondo effect, although there is no static Kondo effect in the ground state (small Fermi surface). Note that the C_{el}-data were obtained[22] with field applied along the crystallographic *ab*-plane. Hence, to enable the comparison made in Supplementary Fig. 8 these data were multiplied by a factor 11 as outlined in Supplementary Note 6.

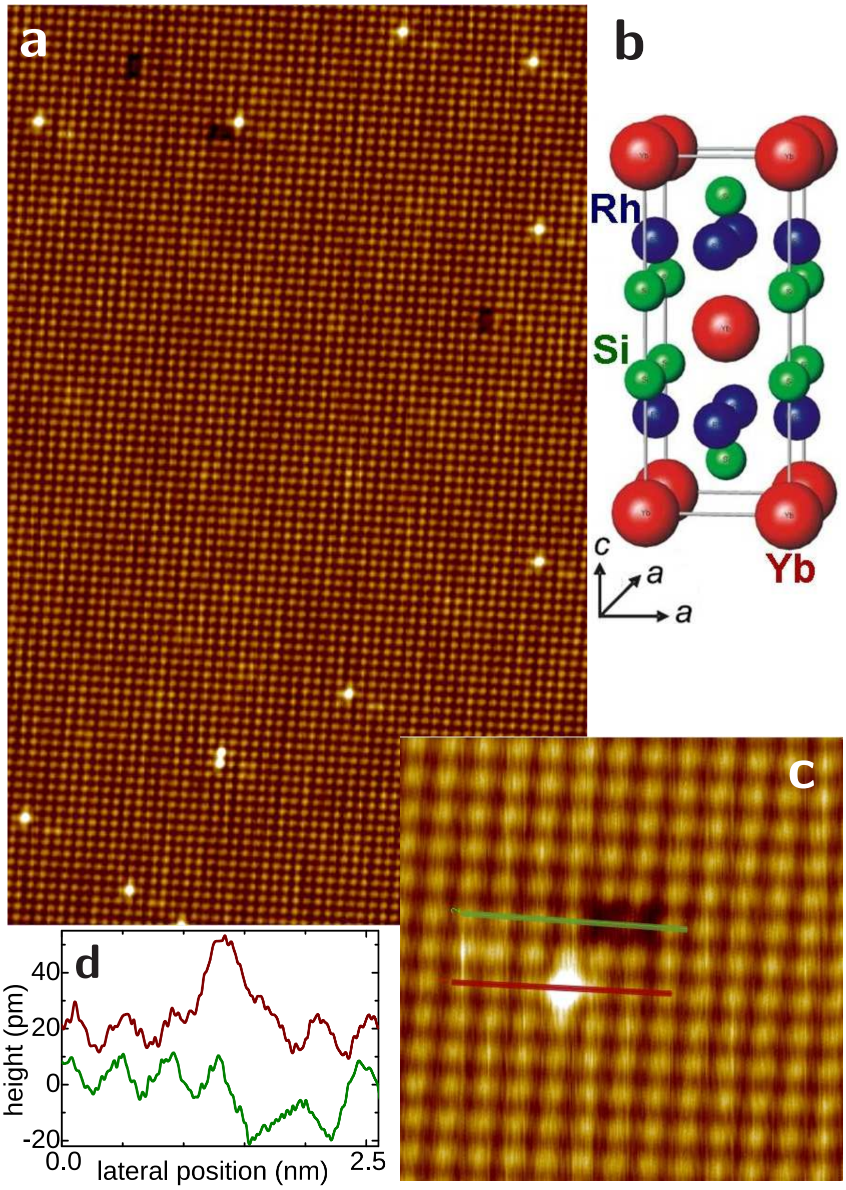

Supplementary Figure 1. **Topography on $YbRh_2Si_2$.** **a**, Topography overview on a $YbRh_2Si_2$ surface. The small number of defects over a large scan area of 20.8×33.2 nm^2 evidences the excellent sample quality. Tunneling parameters: $V = 0.1$ mV, $I = 0.6$ nA. **b**, Tetragonal crystal structure of $YbRh_2Si_2$. **c**, Zoom into an area of 5.2×5.2 nm^2 showing the two types of defects. They indicate a Si-terminated surface. **d**, Height profile along the lines indicated in c), *i.e.* through the defects.

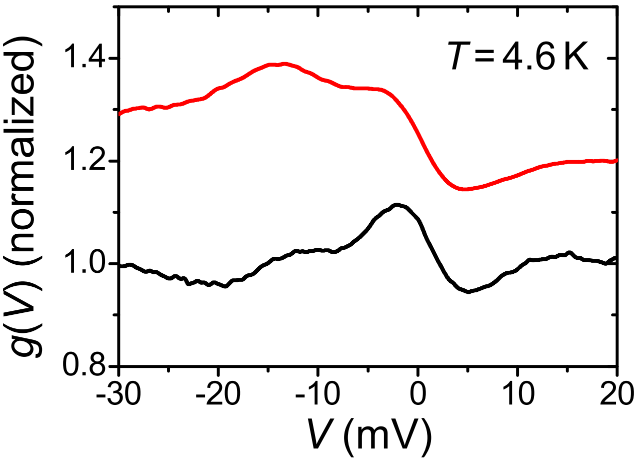

Supplementary Figure 2. **Yb-terminated surface.** Tunneling conductance $g(V, T = 4.6\ \mathrm{K})$ measured at different positions on a Yb-terminated surface.

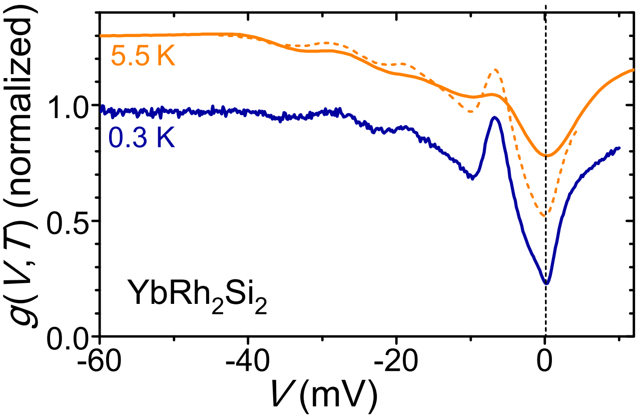

Supplementary Figure 3. **Influence of thermal smearing.** Tunneling conductance $g(V, T)$ measured at $T = 0.3$ K (blue) and 5.5 K (orange). For comparison, the dashed orange line represents the numerical result of the $g(V, T = 0.3\,\mathrm{K})$-curve being thermally smeared to 5.5 K.

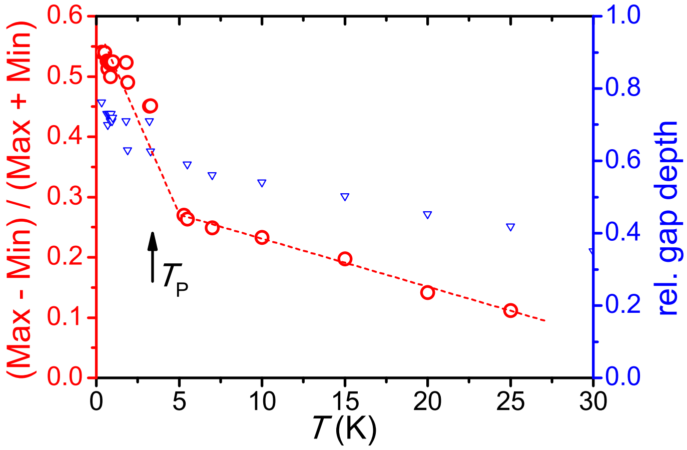

Supplementary Figure 4. **Alternative peak analysis.** Difference of the tunneling conductances $g(V \simeq -6\ \mathrm{meV}, T) - g(V = 0, T)$, *i.e.* at the maximum and the minimum conductance (red circles). For comparison, also the depth of the zero-bias dip is presented (blue markers).

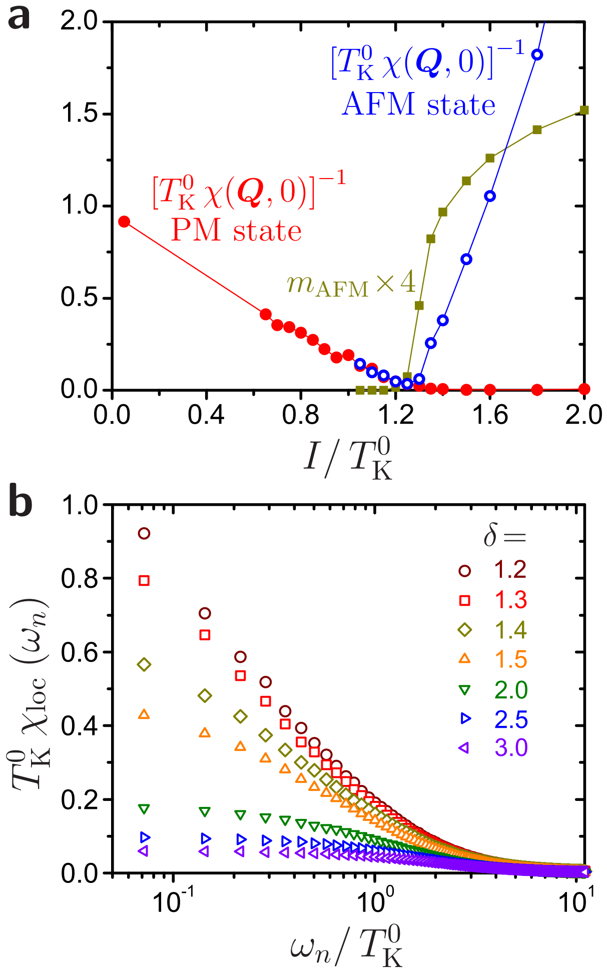

Supplementary Figure 5. **EDMFT analysis of the dynamical Kondo lattice.** **a**, Phase diagram of a Kondo lattice. The tuning parameter is $I/T_{\rm K}^0$, the ratio of the RKKY interaction to the bare Kondo temperature scale. The AFM order parameter, $m_{\rm AFM}$, smoothly sets in as $I/T_{\rm K}^0$ increases through $(I/T_{\rm K}^0)_c \approx 1.2$. The continuous nature of the quantum phase transition is further demonstrated by the vanishing of the inverse static susceptibility at the AFM wavevector $\boldsymbol{Q}$, as $I/T_{\rm K}^0$ approaches $(I/T_{\rm K}^0)_c$ from both sides. **b**, Normalized local dynamical spin susceptibility *vs.* the normalized Matsubara frequency, ω_n, at a low temperature $T \approx 0.01{\cdot}T_{\rm K}^0$. The legend specifies the tuning parameter $I/T_{\rm K}^0$. Figures adapted from Ref. 7.

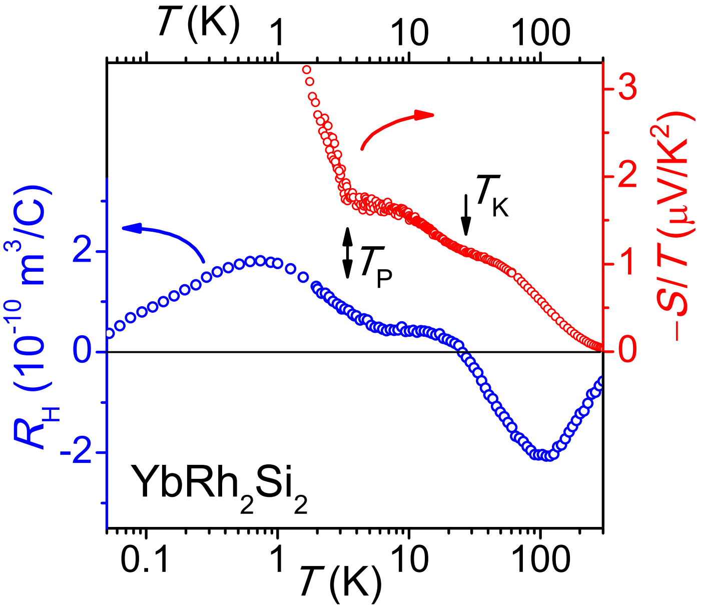

Supplementary Figure 6. **Hall effect and thermopower of YbRh$_2$Si$_2$.** Left: Measured initial Hall coefficient $R_{\rm H}$ (blue circles) in dependence on temperature, from Ref. 11. Note the plateau of R_H between 7 K $\lesssim T \lesssim$ 20 K and and a maximum at around 0.7 K. Right: Thermopower shown as S/T on an enlarged scale (same data as in Fig. 4 of main text). A saturation below about 7 K is disrupted at $T_{\rm P} \cong 3.3$ K by a sudden strong increase of the absolute value of S/T.

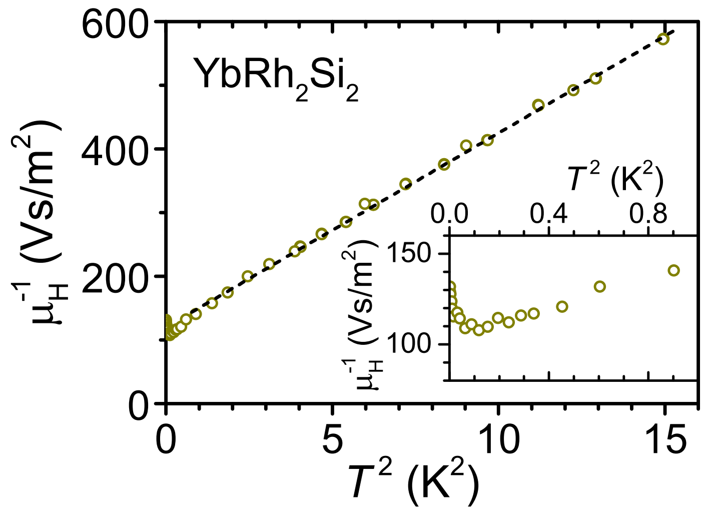

Supplementary Figure 7. **Inverse Hall mobility,** $1/\mu_H = \rho_{xx}/R_H = \cot\theta_H$, in dependence on square temperature, T^2, showing proportionality $1/\mu_H \sim T^2$ within 0.5 K $\lesssim T \lesssim$ 5 K (dashed line). Inset: Zoom into the low-temperature data of $1/\mu_H$.

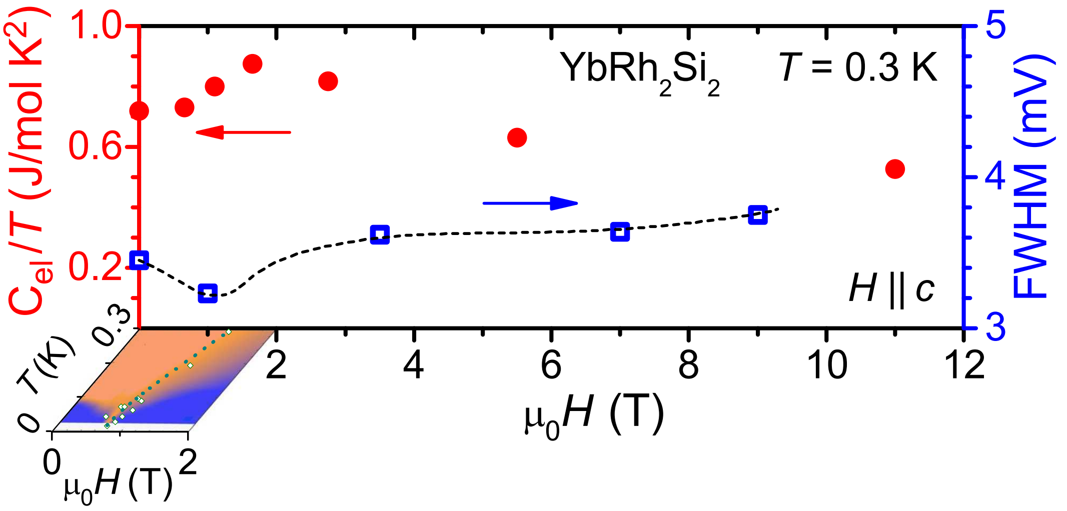

Supplementary Figure 8. **Comparison between STS, specific heat and magnetotransport results.** Magnetic field dependence at $T = 0.3$ K of the electronic contribution to the specific heat plotted $C_{\rm el}/T$ (red dots, left axis), and of the FWHM of the -6 meV-peak seen in STS (blue data, same as in Fig. 5c of the main text). $C_{\rm el}$-values were taken from Refs.[18,22], however with the field values multiplied by a factor 11 to account for the different direction in which the field was applied. Also shown for comparison is the color-coded resistivity exponent ε in $[\rho(T) - \rho(T=0)] \propto T^{\varepsilon}$, where blue regions correspond to $\varepsilon \approx 2$ and orange regions to $\varepsilon \approx 1$ (color-coded Figure adapted from Ref. 13). The shown $T - H$ phase space covers $0 \leq \mu_0 H \leq 2$ T and $0 \leq T \leq 0.3$ K. Hence, the upper T-limit of the presented phase space coincides with the temperature of the FWHM and $C_{\rm el}/T$-plot, allowing direct comparison at this T. The green data mark the line $T_{\rm Hall}$ at which the Hall coefficient exhibits a crossover for $H \parallel c$ (data taken from Ref. 20).

Supplementary References

[1] Krellner, C., Taube, S., Westerkamp, T., Hossain, Z. and Geibel, C. Single-crystal growth of $YbRh_2Si_2$ and $YbIr_2Si_2$. *Phil. Mag.* **92**, 2508-2523 (2012).

[2] Wirth, S., Ernst, S., Cardoso-Gil, R., Borrmann, H., Seiro, S., Krellner, C., Geibel, C., Kirchner, S., Burkhardt, U., Grin, Y. and Steglich, F. Structural investigations on $YbRh_2Si_2$: from the atomic to the macroscopic length scale. *J. Phys.; Condens. Matter* **24**, 294203 (2012).

[3] Ernst, S., Kirchner, S., Krellner, C., Geibel, C., Zwicknagl, G., Steglich, F. and Wirth, S. Emerging local Kondo screening and spatial coherence in the heavy-fermion metal $YbRh_2Si_2$. *Nature* **474**, 362-366 (2011), see Supplementary Information.

[4] Stockert, O., Koza, M. M., Ferstl, J., Murani, A. P., Geibel, C. and Steglich, F. Crystalline electric field excitations of the non-Fermi-liquid $YbRh_2Si_2$. *Physica B* **378**, 157–158 (2006).

[5] Danzenbächer, S., Kucherenko, Yu., Vyalikh, V., Holder, M., Laubschat, C., Yaresko, A. N., Krellner, C., Hossain, Z., Geibel, C., Zhou, X. J., Yang, W. L., Mannella, N., Hussain, Z., Shen, Z.-X., Shi, M., Patthey, L. and Molodtsov, S. L. Momentum dependence of $4f$ hybridization in heavy-fermion compounds: Angle-resolved photoemission study of $YbIr_2Si_2$ and $YbRh_2Si_2$. *Phys. Rev. B* **75**, 045109 (2007).

[6] Güttler, M., Kummer, K., Patil, S., Höppner, M., Hannaske, A., Danzenbächer, S., Shi, M., Radovic, M., Rienks, E., Laubschat, C., Geibel, C. and Vyalikh, D. V., Tracing the localization of $4f$ electrons: Angle-resolved photoemission on $YbCo_2Si_2$, the stable trivalent counterpart of the heavy-fermion $YbRh_2Si_2$. *Phys. Rev. B* **90**, 195138 (2014).

[7] Zhu, J.-X., Grempel, D. R. and Si, Q. Continuous quantum phase transition in a Kondo lattice model. *Phys. Rev. Lett.* **91**, 156404 (2003).

[8] Si, Q., Pixley, J. H., Nica, E., Yamamoto, S. J., Goswami, P., Yu, R. and Kirchner, S. Kondo destruction and quantum criticality in Kondo lattice systems. *J. Phys. Soc. Jpn.* **83**, 061005 (2014).

[9] Glossop, M. T. and Ingersent K. Magnetic quantum phase transition in an anisotropic Kondo lattice. *Phys. Rev. Lett.* **99**, 227203 (2007).

[10] Zhu, J.-X., Kirchner, S., Bulla, R. and Si, Q. Zero-temperature magnetic transition in an easy-axis Kondo lattice model. *Phys. Rev. Lett.* **99**, 227204 (2007).

[11] Friedemann, S., Wirth, S., Oeschler, N., Krellner, C., Geibel, C., Steglich, F., MaQuilon, S., Fisk, Z., Paschen S. and Zwicknagl, G. Hall effect measurements and electronic structure calculations on $YbRh_2Si_2$ and its reference compounds $LuRh_2Si_2$ and $YbIr_2Si_2$. *Phys. Rev. B* **82**, 035103 (2010).

[12] Paschen, S., Lühmann, T., Wirth, S., Trovarelli, O., Geibel, C. and Steglich, F. Anomalous Hall effect in $YbRh_2Si_2$. *Physica B* **359-61**, 44–46 (2005).

[13] Custers, J., Gegenwart P., Wilhelm, H., Neumaier, K., Tokiwa, Y., Trovarelli, O., Geibel, C., Steglich, F., Pépin C. and Coleman, P. The break-up of heavy electrons at a quantum critical point. *Nature* **424**, 524–527 (2003).

[14] Hartmann, S., Oeschler, N., Krellner, C., Geibel, C., Paschen, S. and Steglich, F. Thermopower evidence for an abrupt Fermi surface change at the quantum critical point of $YbRh_2Si_2$. *Phys. Rev. Lett.* **104**, 096401 (2010).

[15] Friedemann, S., Oeschler, N., Wirth, S., Krellner, C., Geibel, C., Steglich, F., Paschen, S., Kirchner, S. and Si, Q. Fermi-surface collapse and dynamical scaling near a quantum-critical point. *Proc. Natl. Acad. Sci. USA* **107**, 14547-51 (2010).

[16] Pfau, H. *et al.* Interplay between Kondo suppression and Lifshitz transitions in $YbRh_2Si_2$ at high magnetic fields. *Phys. Rev. Lett.* **110**, 256403 (2013).

[17] Naren, H. R., Friedemann, S., Zwicknagl, G., Krellner, C., Geibel, C., Steglich, F. and Wirth, S. Lifshitz transitions and quasiparticle de-renormalization in $YbRh_2Si_2$. *New J. Phys.* **15**, 093032 (2013).

[18] Trovarelli, O., Geibel, C., Mederle, S., Langhammer, C., Grosche, F. M., Gegenwart, P., Lang, M., Sparn, G. and Steglich, F. $YbRh_2Si_2$: Pronounced non-Fermi-liquid effects above a low-lying magnetic phase transition. *Phys. Rev. Lett.* **85**, 626-629 (2000).

[19] Custers, J., Gegenwart, P., Geibel, C., Steglich, F., Tayama, T., Trovarelli, O., Harrison, N. Low-temperature magnetic and transport properties of the clean NFL system $YbRh_2(Si_{1-x}Ge_x)_2$. *Acta Phys. Pol. B* **32**, 3211–3217 (2001).

[20] Paschen, S., Lühmann, T., Wirth, S., Gegenwart, P., Trovarelli, O., Geibel, C., Steglich, F., Coleman, P. and Si, Q. Hall-effect evolution across a heavy-fermion quantum critical point. *Nature* **432**, 881–885 (2004).

[21] Gegenwart, P., Westerkamp, T., Krellner, C., Tokiwa, Y., Paschen, S., Geibel, C., Steglich, F., Abrahams, E. and Si, Q. Multiple Energy Scales at a Quantum Critical Point. *Science* **315**, 969-971 (2007).

[22] Oeschler, N., Hartmann, S., Pikul, A. P., Krellner, C., Geibel, C. and Steglich F. Low-temperature specific heat of $YbRh_2Si_2$. *Physica B* **403**, 1254-1256 (2008).
